# Case-control study of human anthrax outbreak investigation in farta woreda, South Gondar, Northwest Ethiopia

**DOI:** 10.1186/s12879-023-08136-9

**Published:** 2023-03-17

**Authors:** Taddie Wondmnew, Biset Asrade

**Affiliations:** 1 Field Epidemiology, School of Public Health, Addis Ababa University, Addis Ababa, Ethiopia; 2 Department of Pharmacy, College of Medicine and Health Sciences, Bahir Dar University, Bahir Dar, Ethiopia

**Keywords:** Anthrax, Confirmed, Outbreak, Northwestern Ethiopia

## Abstract

**Background:**

Anthrax is a zoonotic disease caused by the *Bacillus anthracis* bacteria, which is one of the top five important livestock diseases and the second top priority zoonotic disease, next to rabies, in Ethiopia, which remains a major problem for animals and public health in Ethiopia. This study was conducted to verify the existence of the outbreak, determine risk factors, and implement measures to control the anthrax outbreak in Farta woreda, South Gondar zone, Northwest Ethiopia in 2019.

**Methods:**

A community-based case-control study was conducted from March 25 to April 1, 2019. A structured questionnaire was used to collect data and for review of documents and discussion with livestock and health office staff. The collected data were analyzed by SPSS and presented in tables and graphs.

**Results:**

A total of 20 human anthrax cases with an attack rate of 2.5 per 1000 population were reported from the affected kebele. The age of the cases ranged from 1 month to 65 years (median age = 37.5 years). Of the total cases, 66.7% were male and 77.8% were 15 and older. The probability of developing anthrax among people who had unvaccinated animals was higher than in those who didn’t have unvaccinated animals with an AOR = 8.113 (95% CI 1.685–39.056) and the probability of getting anthrax in relation to people’s awareness of anthrax was AOR = 0.114 (95% CI 0.025–0.524).

**Conclusion:**

An anthrax outbreak occurred in Wawa Mengera Kebele of Farta woreda. The presence of unvaccinated animals in a household was found to be a risk factor for anthrax cases. Timely animal vaccination and strengthening health education on the vaccination of animals, mode of transmission, and disposal of dead animals are essential for preventing anthrax cases.

## Background

Anthrax is an acute disease of warm-blooded animals, including human beings, caused by spore-forming gram-positive, non-motile *bacillus anthracis* [1]. The name of the bacterium is derived from “*anthrakis”*, the Greek word for coal, because anthrax in humans causes black, coal-like lesions on the skin at the site of inoculation [2].

Anthrax is a zoonotic bacterial disease caused by *Bacillus anthracis*, which primarily affects herbivorous wildlife and livestock and is usually fatal among these animals. Zoonoses are diseases that are transmissible between animals (domestic and wildlife) and humans. It has been estimated that 60% of all human diseases and around 75% of emerging infectious diseases are zoonotic, among which anthrax is a serious disease that can affect most mammals and several species of birds [3].

Anthrax is an important but neglected zoonosis in many parts of the world. The disease is mainly endemic in developing countries [3, 4].

In Ethiopia, anthrax is endemic and occurs before and after the rainy season. Most commonly, anthrax cases are reported in areas with high levels of salt soil [5]. It is one of the top five important livestock diseases and the second top priority zoonotic disease, next to rabies, in Ethiopia, which remains a major problem for animals and public health in Ethiopia. Particularly, the Amhara Regional state is frequently affected by diseases due to a humid, sub-humid environment, weak animal health services, and a lack of awareness of the community about animal anthrax case management, which leads to widespread outbreaks [6–8].

In Ethiopia, from July 2016 to January 30/2019, according to Ethiopian Public Health Institute (EPHI) and Public Health Emergency Management (PHEM) surveillance data, a total of 1,188 suspected human anthrax cases and 15 deaths with a case fatality rate (CFR) of 1.3%, were reported from four regions (Tigray, Amhara, Oromia, and South Nation nationalities of people (SNNPR). The highest number of cases were reported from Amhara (816), followed by Tigray (250), SNNPR (89), and Oromia (32). The highest number of deaths [9] was reported in Amhara (67% of the total deaths), with Oromia and Tigray having 2 deaths each (13.3%), and SNNPR one death (6.7%) [10].

Although suspected cases of anthrax in livestock are reported from Farta woreda, few are officially confirmed via polymer chain reaction (PCR) [10]. Previous studies in Ethiopia indicate that the disease is well recognized by rural communities but little is known about its prevalence, epidemiology, and public health significance (11.

The most efficient ways of preventing and controlling anthrax infection in domestic herds are sustainable surveillance, annual vaccination of livestock, and proper carcass disposal management [6–10].

### Justification of the study

**Fig. 1:**
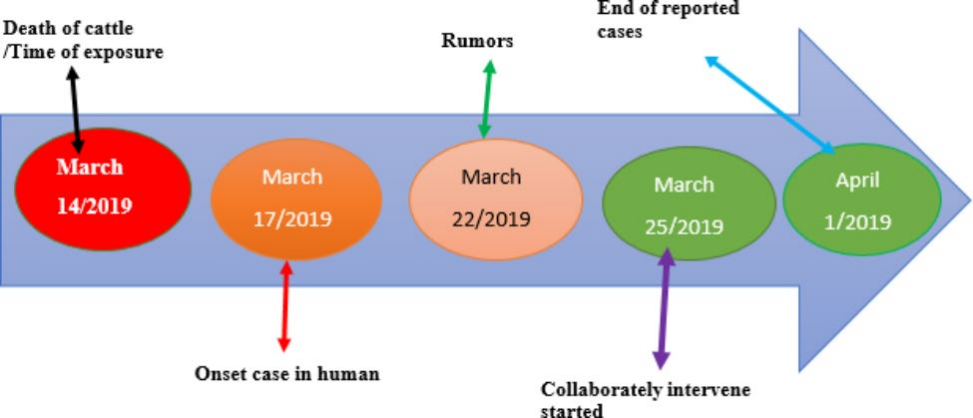
depicts the progression of the anthrax situation in Farta woreda, South Gondar zone, Northwestern Ethiopia, from the time of cattle exposure to the end of cases reported in 2019

On March 23, 2019, suspected human anthrax cases and the deaths of animals were captured from social media (Facebook) scanning. To verify this rumor, the Amhara Public Health Institute communicated with the South Gondar zone health department officers and confirmed that two suspect cases of human anthrax were admitted to Debre Tabor General Hospital and an additional two animal deaths were reported from the Farta district. Simultaneously, we communicated with the Ethiopian Public Health Institute for further confirmation of cases through the testing of clinical specimens (Fig. 1). They collected blood and tissue scraps from suspected human anthrax cases.

Outbreak investigations of anthrax are important for strengthening the surveillance system, clarifying gaps, identifying anthrax cases early, and strongly promoting a one-health approach. Therefore, this study addresses the root causes of the anthrax outbreak and identifies anthrax cases for the implementation of prevention and control activities in the South Gondar zone, prioritizing anthrax as a public health threat for the time being. The objectives of this study were to verify the existence of outbreaks and identify risk factors for anthrax in Farta woreda, South Gondar zone, Amhara region, in March 2019.

## Methods and materials

### Study area

Farta is one of the 18 woredas in the South Gondar zone and has 32 kebeles. The investigation was conducted in Wawa Mengera kebele of Farata woreda, south Gondar zone, Amhara region.

### Study Design

An unmatched case-control investigation was conducted in Farta woreda of the South Gondar zone, Northwestern Ethiopia.

### Study period

An anthrax outbreak investigation was conducted from March 25-April 1, 2019.

### Sample size determination

The sample size was determined using Epi Info**™** 7 statistical tools, but due to the small number of human anthrax cases in Wawa Mengera Kebele, all 20 human anthrax cases were included. Controls were selected by neighborhood approach from human anthrax cases with the same exposure status.

### Dependent variable

Cases and controls of study participants are dependent variables.

### Independent variables

Study participants’ ages, sexes, and the presence of unvaccinated cattle in the household were independent variables.

## Operational definition

### Cutaneous

skin lesion evolving over 1 to 12 days from a papule to a vesicle, to a depressed black eschar, invariably accompanied by edema that may be mild to extensive.

### Inclusion criteria from cases

All human anthrax cases in Wawa Mengera Kebele of Farta woreda, South Gondar zone, Northwestern Ethiopia between March 25 and April 1, 2019.

### Exclusion criteria from cases

People who had mechanical injuries or warts leading to wounds in their bodies didn’t have cutaneous anthrax and people who had cutaneous anthrax coming from other woredas aren’t abused as the result of the study.

### Data collection procedure and tool

Cases were reported up to April 1, 2016, which became a high number on March 20, 2019, based on our investigation and interviews there. Public health intervention strategies began on March 23, 2019. The number of cases decreased after burned and buried meats were properly disposed of in their homes.

We collected data from cases and controls by moving house to house in the exposed villages from March 25–30, 2019. Participants were interviewed using structured questionnaires and primary data were collected from respondents by the investigator. The cases were identified using a case definition. If an identified case patient was unable to respond to the questionnaire, an attendant in the household provided the data. A total of 20 cases and 40 controls were interviewed to assess their exposure status, sociodemographic, and other variables.

### Cases

Persons who had lesions in their body parts, whether they participated in slaughtering or not, but only presented in this affected Kebele/village.

### Controls

Persons who participated in the slaughter of dead animals but did not have anthrax-infected people in their homes or did not exhibit signs and symptoms of cutaneous anthrax were used as controls. The controls were based on an unmatched neighborhood approach.

### Data processing and analysis

Excel Office 2013 was used to clean, enter, and analyze the data. Descriptive statistics were used to create a profile of sociodemographic characteristics, the history of the area for anthrax, and the boundaries of the endemic for both human and animal anthrax cases. Finally, the results were presented with tables, graphs, and figures.

## Results

### Sociodemographic of Farta woreda

Farta woreda is a woreda of the South Gondar zone. The community comprised a mixed farm system (rearing livestock and cultivation). The woreda contains traditional hide and skin processors, which are found in Wawa Mengera Kebele. Around the traditional hide processors, one river flows down to the east and has a history of anthrax cases around the site. The study area is highland and has high rainfall. Eventually, soil pH of the area is acidic (5.3–6.1) as I gathering the result from Amhara Design and regulatory enterprise.

### Description of the outbreak by person

**Fig. 2:**
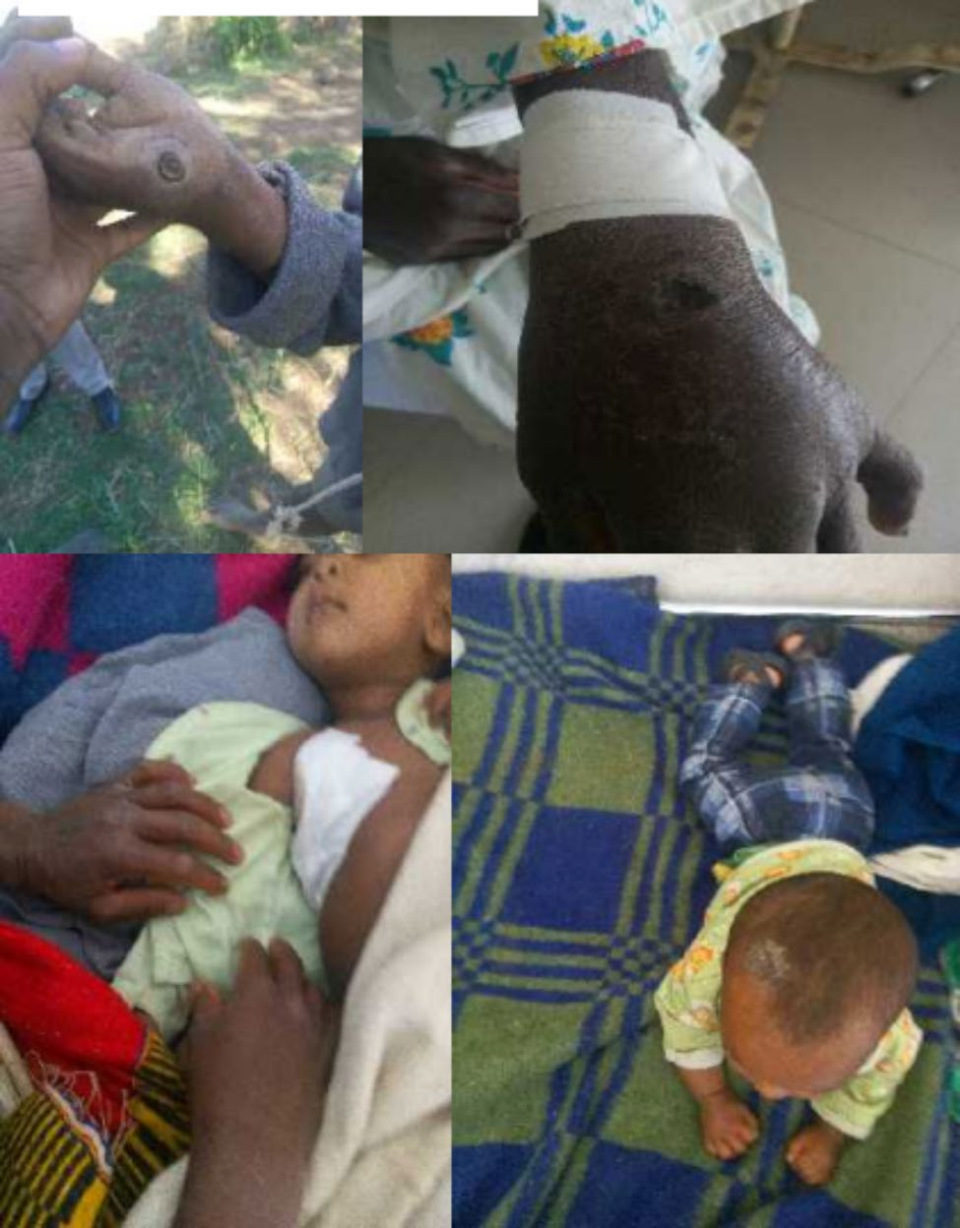
Photographs taken during outbreaks of anthrax in Farta woreda, South Gondar zone, Northwestern Ethiopia, 2019

Until this investigation was completed, 20 cases were discovered. The most affected age group was the greater than or equal to 15 age categories (80%), while the least affected was the 0–4 age category (10%) of the total study participants. All of the case patients presented with cutaneous lesions on their hands or upper neck (Fig. 2). Cases aged from 0 to 14 (20%) were affected only in the upper neck (Table 1).

**Table 1:** Demographic data of Anthrax outbreak study participants in the study area

Variable	Categories	Cases (N = 20)	Percentage (%)	Controls (N = 40)	Percentage (%)
Gender	Male	14	70	27	67.5
	Female	6	30	13	32.5
Marital status	Married	13	65	25	62.5
	Single	4	20	9	22.5
	Illegible	3	15	6	15
Age	0–4 age	2	10	3	7.5
	5–14 age	2	10	5	12.5
	≥ 15 age	16	80	32	80
Occupation	Farmers	13	65	25	62.5
	Students	5	25	11	27.5
	Unable to	2	10	4	10

### The description of the outbreak by place

The index exposure/common source of the cases was a cow that died of anthrax in Wawa Megara kebele, which confirmed a case of anthrax from a meat sample that had a positive test result. The raw meat of the dead cows was distributed in three gots (villages). Human anthrax cases were distributed throughout multiple gots, including Mandera (9/50%), Lemo (3/16.7%), and the rest of the multiple gots. Only one was identified as having animals affected by anthrax. The locations of the affected villages are near the local hide processor villages and there is a water source for animal feed purposes.

### The description of the outbreak by time

**Fig. 3:**
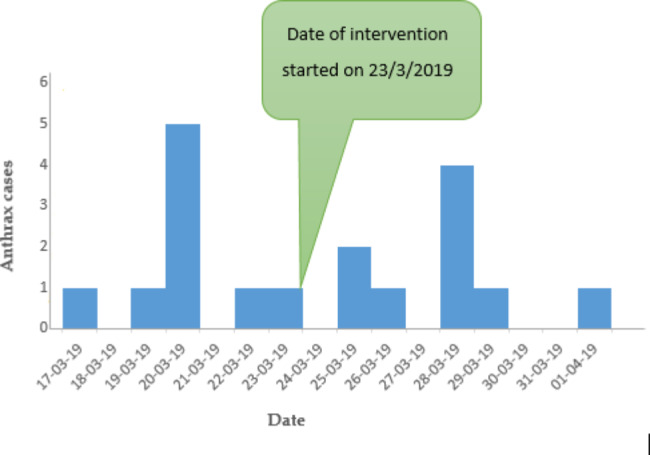
Epi curve of the anthrax outbreak in Farta woreda, South Gondar zone, Northwestern Ethiopia, 2019

The first human case in Mandera occurred on March 17, 2019, following the death of three animals in Wawa Mengera kebele (Mandera) without having vaccination history. A total of four animals died. The raw meat of the cow that died first was distributed to different villages. The sequences of events of the outbreak from the beginning to the end were illustrated using Epi Curve (Fig. 3).

### Risk factors for contracting anthrax

A person who possesses a hide/skin taken from other areas and after 3 days the grazing cow and sheep in their facing area was dead the, a cat ate lung of cow. All cases who participated in the study directly or indirectly had contact with carcasses of dead cows or their products during the different activities of processing the meat and hide. Only 11 people were involved in butchering and preparing hides for further processes and we addressed 60 households (247 people) in the study area who have direct or indirect contact with raw meat, and no one ate the raw meat.

## Bivariate analysis

Because of the initial case was grazing around local skin-possessing villages with unvaccinated history, others grazing there with vaccinated history not dead or sign of infection. People who had unvaccinated cattle during the campaign were also a risk factor for OR = 6.79 (95% CI 1.911–2.908). The probability of getting anthrax in relation to people’s lack of awareness about anthrax was a risk factor OR = 5.909 (95% CI 1.6920.659). (Table 2).

**Table 2:** The result is a bivariate analysis of variables in human anthrax cases in the study area

Variables	Category	Cases (N = 20)	Control (N = 40)	AOR (95% CL) *	P-value	
Did you have any awareness about anthrax before this outbreak?	Yes	7(35%)	9(22.5)	5.909(0.1.690- 20.659)	0.005	
	No	13(65%)	31(77.5%)			
Having unvaccinated domestic animals	Yes	9(45%)	27(67.5%)	6.760(1.911–23.908)	0.003	
	No	13(55%)	13(32.5%)			

*AOR = Adjusted Odds Ratio; CI = Confidence interval at 95% significance level.

### Multivariate analysis

People who had unvaccinated domestic animals during the campaign and routine immunization program (AOR = 8.113 (95% CI 1.685–39.056)) were independent risk factors.

for anthrax in Wawa Mengera kebele, Farta woreda. Participants in the study who had heard about anthrax disease (AOR = 0.114 (95% CI 0.025 0.524) (Table 3).

**Table 3:** The result of the multivariate analysis of human anthrax cases is significantly associated with factors in the univariate analysis

Variables	Category	Cases (N = 20)	Control (N = 40)	AOR (95% CL)	P-value
Have you heard about anthrax?	Yes	7(35%)	9(22.5)	0.114(0.025–0.524)	0.005
	No	13(65%)	31(77.5%)		
Having unvaccinated domestic animals?	Yes	9(45%)	27(67.5%)	8.113(1.685–39.056)	0.009
	No	13(55%)	13(32.5%)		

### Knowledge of signs and symptoms of anthrax in humans

From control study participants, 25 (69.4%) have awareness about anthrax before the outbreak. With regard to signs and symptoms, all cases showed typical anthrax skin lesions, and six cases were hospitalized for 7 to 14 days at Debre Tabor General Hospital. A single person who has butchered a dead cow: - first suffered from cutaneous once like others after a time became a meningitis anthrax form with a 14–17 day stay at ICU with the help of an NG tube. Not rolling of their neck spastic of over shoulder muscles. After a 7-day vancomycin Rx,there were some signs.

### Symptoms experienced and the treatment-seeking behavior of respondents

Most cases, 12 (66.7%), received health care services in government health facilities, both inpatient and outpatient conditions, and the rest in private health facilities. The majority, 15 (83.3%) cases, took ciprofloxacin treatment, whereas the rest were treated with clindamycin from private health facilities.

### Attitude and practices regarding the disposal of dead animals

Two dead animals were buried before being opened and two tested positive after being opened.

### Laboratory results

Both human sludges touched and scraped at the infection site samples and animal meat tested positive in molecular tests. Animal dry meat was tested at the end of march 2019 at the national animal health diagnosis and investigation center by polymerase chain reaction (PCR) and human samples were tested at the Ethiopia Public Health Institute (EPHI).

### Public health measure

After scanning for anthrax cases on social media, the South Gondar zonal health department, the Farta woreda health office, the South Gondar zonal livestock department, and the Farta woreda livestock officers discussed and re-established rapid response teams (RRTs).

## Discussion

The intervention measures included cattle vaccination, treatment of cases, health education to the community, burning, and burying of infected meat and carcasses, among others.

**On multivariate analysis**, risk factors were the presence of unvaccinated cattle in their house, OR = 8.113 (95% CI 1.685–39.056). This might be due to the incision of the animal when they showed signs and symptoms before taking veterinary clinics. Among the interviewed case respondents, 8 infected people incised their animals during the time of the outbreak when the animal showed clinical signs of swelling before they visited veterinary clinics.

The position of the most anthrax lesions were on the hands of case patients older than 10 years and younger than 10 years. This is consistent with Gombe et al.’s findings from a study in Zimbabwe [11]. This may be because hands are actively involved in handling the infected meat and may have a higher risk of having cuts or open wounds, which increases the likelihood of infection in that part of the body [11].

No case fatalities were recorded in this outbreak. This is consistent with the findings by Gombe et al. in Zimbabwe [11]. A person who has heard of what anthrax is before the outbreak had an AOR = 0.114 (95% Cl 0.025-0524), which means a protective factor for anthrax cases compared to those who haven’t heard of anthrax cases. This might be because they restricted themselves or properly handled infected animals from contact with infected animals and animal products during the outbreak period.

Having cuts or open wounds during processing in areas likely to come into contact with infected material increases the risk of anthrax. This was also because these areas were unobstructed routes of entry for large numbers of bacteria compared with intact skin. This was consistent with what Gombe et al. found in an anthrax outbreak in Zimbabwe [11].

The strength of the investigation was that it increased interaction and information sharing between veterinarians and human health workers. Immediately after the investigation, we conducted a workshop on zoonotic diseases, including anthrax and rabies, with participants from the environment, forest and wildlife authorities, animal health workers, and human health workers. This investigation was an initiative of a one-health approach in the Amhara region. Having positive laboratory results for animal samples.

### Limitations of the study

This investigation only focused on human anthrax cases and the absence of cattle vaccination data for each species. The case sample size is small, and the investigation is limited in time.

## Conclusion and recommendations

Unvaccinated histories of dead animals were the source of human anthrax in Wawa Mengera Kebele of Farta woreda. The overall attack rate was 2.5 per 1000 people in the affected kebele. Complicated anthrax cases have occurred because of recent visits to health facilities. People who have unvaccinated animals are a risk factor for the occurrence of anthrax cases. People who had heard of anthrax before outbreaks, on the other hand, are protective factors against anthrax outbreaks due to their limited contact with dead animals and their products.

Based on the findings, the following recommendations are made: [1] timely animal vaccination; [2] strengthening health education on how Anthrax is transmitted from livestock to humans; [11] health and livestock offices advocate avoiding properly disposed dead animals, skinning, and hide use; and [3] strengthening a single health approach to intervene early in zoonotic disease.

## Data Availability

All the data which were used for this study are not available in the public domain, but anyone interested in using the data for scientific purposes is free to request permission from the corresponding author via email: biset2006me@gmail.com.

## Declarations

We obtained written informed consent from all study participants above 16 years of age and from their parents of the participants below 16 years of age before data collection. The data collection was started after getting permission and provision in accordance with the relevant guidelines. The study was conducted in alignment with home case treatment and prevention activities.

## Abbreviations

CDC: Center of Disease Control and Prevention
EFETP: Ethiopian Field Epidemiology Training Program
EPI: Expanded Program on Immunization
FMoH: Ethiopian Federal Ministry of Health
WHO: World Health Organization
